# Prophylactic transcatheter arterial embolization for high-risk ulcers following endoscopic hemostasis: a meta-analysis

**DOI:** 10.1186/s13017-021-00371-2

**Published:** 2021-06-10

**Authors:** Qian Yu, Chenyu Liu, Biagio Collura, Rakesh Navuluri, Mikin Patel, Zhiyong Yu, Osman Ahmed

**Affiliations:** 1 Shandong Cancer Hospital and Institute, Shandong First Medical University and Shandong Academy of Medical Sciences, Jinan City, Shandong Province China; 2 Department of General Surgery, Cleveland Clinic Florida, Florida, USA; 3 School of Medicine, George Washington University, Washington DC, USA; 4 School of Medicine, Ross University, Miramar, FL USA; 5 Division of Interventional Radiology, Department of Radiology, University of Chicago, Chicago, IL USA; 6 Department of Radiology, University of Arizona Medical Center, Tucson, AZ USA

**Keywords:** Embolization, Gastrointestinal bleeding, Interventional radiology, Ulcer

## Abstract

**Background:**

To conduct a meta-analysis to assess the safety and efficacy of prophylactic transcatheter arterial embolization (PTAE) for the treatment of high-risk bleeding peptic ulcers after achieving endoscopic hemostasis.

**Methods:**

PubMed and Cochrane Library were queried for full-text articles published up to December 2019. The following keywords were used: “prophylactic embolization”, “supplement embolization”, “gastrointestinal bleeding”, and “ulcer bleeding”. High-risk ulcers were defined based on endoscopic findings (i.e., large ulcers, Forrest class I-IIb) and/or clinical presentation (i.e., hypotension, decreased hemoglobin during endoscopy). Only comparative studies investigating PTAE versus conservative treatment after achieving endoscopic hemostasis were included. Baseline study characteristics, rebleeding rate, need for surgery, mortality, and PTAE-related complication rates were investigated. Quantitative analyses were performed with Stata 15.1.

**Results:**

Among the five included original studies, a total of 265 patients received PTAE and 617 were managed conservatively after endoscopy. The rebleeding rate (6.8% vs 14.3%, *p* = 0.003) and mortality (4.5% vs 8.8%, *p* = 0.032) of patients from the PTAE group were lower than the control group. PTAE also reduced the cumulative need for future surgical intervention (3.0% vs 14.4%, p = 0.005). The PTAE-related major and minor events were 0.75% and 14.4%, respectively.

**Conclusion:**

PTAE had therapeutic potentials in reducing rebleeding risk, need for surgical intervention, and morality in high-risk peptic ulcers after achieving endoscopic hemostasis. The embolization-associated adverse events were minimal. Future studies should aim to increase the sample size and resources for performing endovascular interventions.

**Supplementary Information:**

The online version contains supplementary material available at 10.1186/s13017-021-00371-2.

## Introduction

In the USA, acute nonvariceal upper gastrointestinal bleeding (UGIB) occurs at an incidence rate of 160 per 100,000 people and represents an estimated seven billion dollars of the annual economic healthcare burden [1, 2]. Furthermore, the mortality rate for UGIB remains as high as 14% despite aggressive medical interventions [3]. Currently, endoscopic interventions such as clipping, thermal coagulation, and epinephrine injection remain the first-line standard [4–6]. Despite successful endoscopic hemostatic control and adjunctive anti-acid treatment, some ulcers (i.e., hemodynamically unstable patients, larger ulcers, etc.) remain at high risk for rebleeding [7–9], leading to increased mortality compared to initial events [10]. Prior retrospective studies have explored the use of prophylactic transcatheter arterial embolization (PTAE) in this patient population to decrease the rebleeding risk, demonstrating promising results [11–13].

In a recently published randomized-controlled trial (RCT), Lau et al. evaluated the efficacy of PTAE after successful endoscopic control of UGIB [8]. No statistically significant difference was observed between the outcomes of patients who received PTAE compared to patients who received endoscopy only (EO) with no further intervention, as measured by 30-day rebleeding, mortality, and re-intervention rates. However, as noted by Loffroy et al. in a response letter to the editor, a large majority of rebleeding and deaths were patients allocated to the PTAE group who failed to receive the intervention [14]. Furthermore, the lack of statistical significance may be attributed to underpowering of the prior studies. In order to overcome this limitation, the purpose of this study was to conduct a meta-analysis of previously published literature to evaluate the safety and efficacy of PTAE in preventing rebleeding after successful endoscopic control of high-risk arterial UGIB.

## Materials and methods

### Literature search

This meta-analysis complied with the Preferred Reporting Items for Systematic Reviews and Meta-analysis Statement [15]. PubMed and Cochrane Library were queried to identify peer-reviewed articles concerning prophylactic embolization of high-risk peptic ulcers versus no intervention after endoscopic hemostasis control. High-risk ulcers were defined based on endoscopic findings (i.e., Forrest class I-IIb) and/or clinical presentations at the operator’s discretion (i.e., hypotension, low hemoglobin during endoscopy, systemic anticoagulation, thrombocytopenia, challenging endoscopic hemostasis, etc.). All databases were queried from their establishment to December 2019. The following keywords were used: “prophylactic embolization”, “supplement embolization”, “gastrointestinal bleeding”, and “ulcer bleeding”.

### Inclusion criteria and exclusion criteria

The following inclusion criteria were adopted: (a) population, patients with arterial UGIB; (b) diagnosis, endoscopy visualization; (c) treatment, PTAE for endoscopically treated bleeding; (d) outcomes, mortality, rebleeding, need for subsequent surgical intervention, PTAE-related complications; and (e) study design, comparative studies of PTAE versus no further intervention (endoscopy only, EO) after endoscopic management. A study was excluded if any of the following criteria were met: (a) non-human studies; (b) sample size less than five; (c) letters, editorials, commentaries, reviews, or case reports; (d) duplicated or patient samples used by more than one study; and (e) non-comparative studies.

**Fig. 1:**
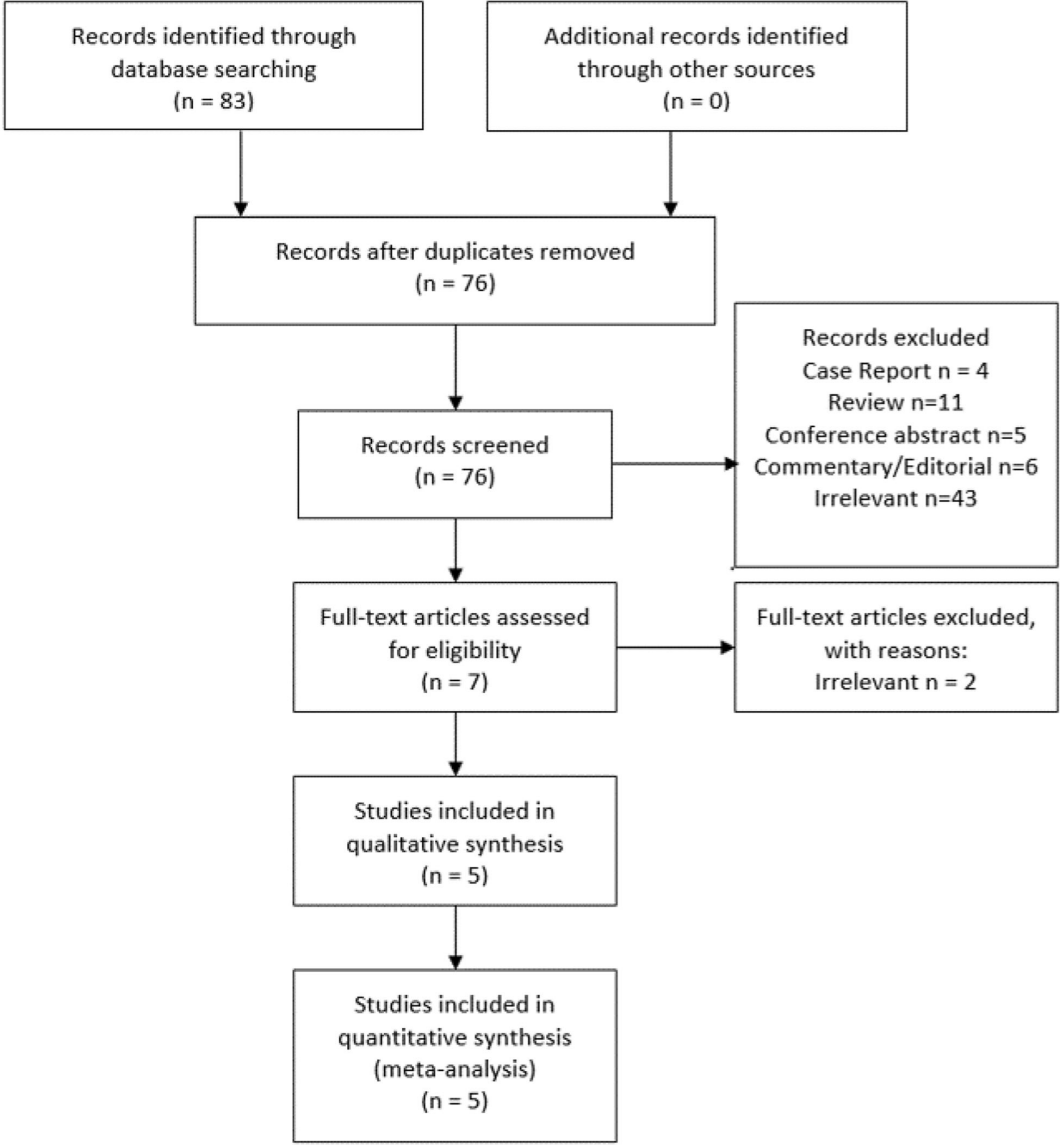
Flow-diagram of the screening process

Endnote X8 (Clarivate Analytics, Philadelphia, Pennsylvanian) was used to identify and delete duplicates. Titles, abstracts, and keywords were screened, followed by the review of full texts of the remaining studies. A detailed screening process is depicted in Fig. 1.

### Data collection and statistical analysis

Baseline characteristics were extracted from each study including author, year of publication, country, study design, sample size, Forrest classification, indication of PTAE, rebleeding rate, mortality rate, need for surgery, and PTAE-related complications. Quality assessment was performed using the Cochrane Collaboration’s tool for randomized-controlled trials and Newcastle Ottawa Scale for cohort studies [16, 17]. Two researchers screened and extracted the data from the original studies. Any disagreement was discussed and arbitrated by a third author if not resolved.

Quantitative analysis was performed with Stata 15.1 (STATA Corp., College Station, TX, USA). Meta-analysis was conducted with the -*metan* function. A fixed-effect model was implemented if heterogeneity I^2^ < 50. A random-effect model was used if I^2^ > 50. Outcomes were pooled if reported by original articles. Overall rebleeding, mortality, and need-of-surgery rates were calculated by dividing the cumulative number of events by the total number of patients from each study. Publication bias was evaluated with funnel plot and Egger’s test. Sensitivity analysis was performed with the one-study remove approach (metaninf analysis).

## Results

### Baseline characteristics of the included studies

After duplicated studies were removed, a total of 76 studies underwent the screening process. Excluded studies included 4 case reports, 11 review papers, 5 conference abstracts, 6 commentaries or editorials, and 43 irrelevant studies. Next, two irrelevant articles were removed upon full-text assessment. Finally, five unique studies were included in the meta-analysis.

**Fig. 2:**
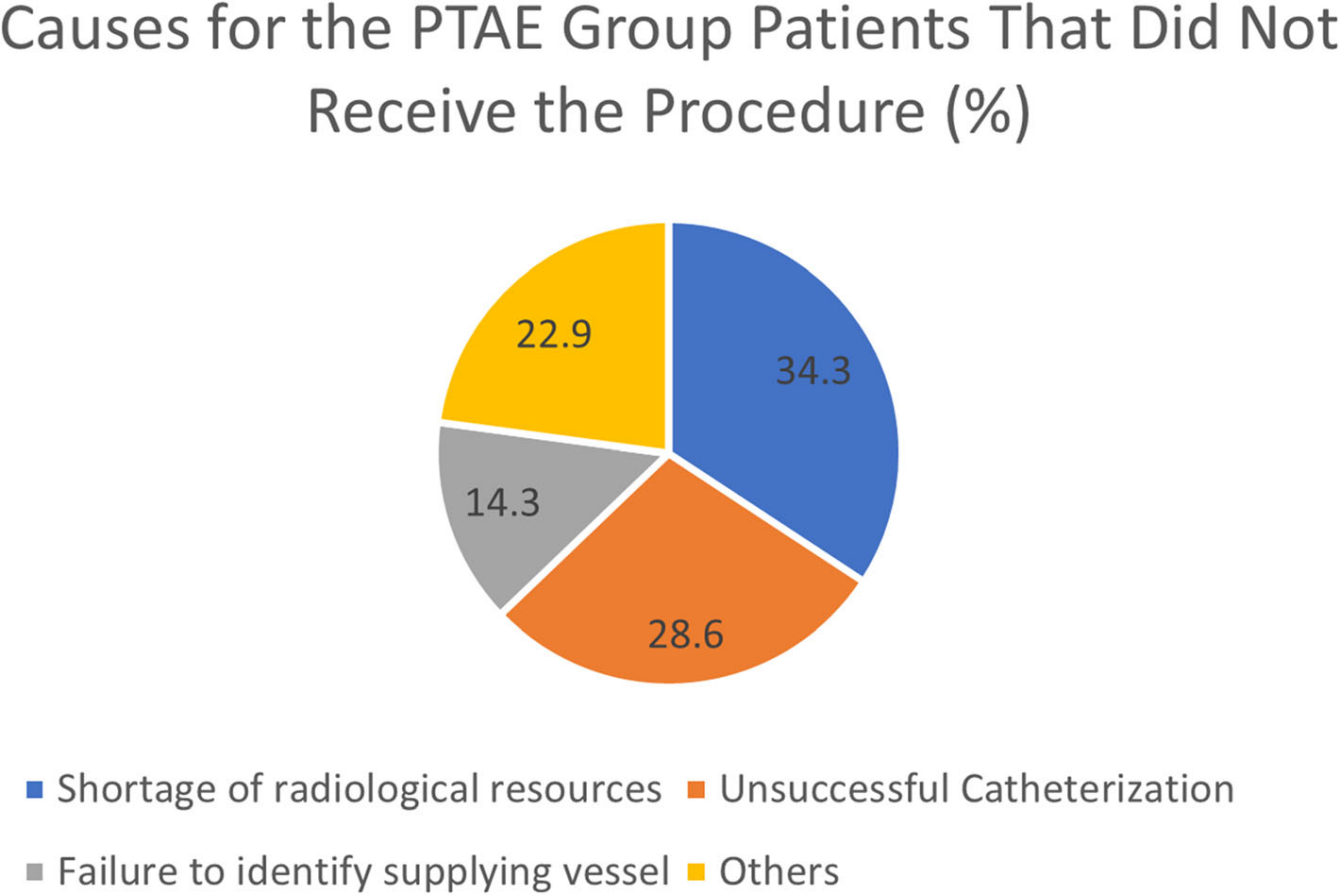
Reasons for failure to receive embolization among patients allocated to PTAE

A total of 882 patients were included [8, 11–13, 18]: 265 and 617 patients received PTAE and endoscopy only, respectively (Table 1). Two studies were randomized-controlled trials (RCTs), and these studies provided 127 PTAE patients and 179 EO patients [8, 18]. The remaining three studies were retrospective cohort (RC) studies that contributed 138 PTAE patients and 438 EO patients [11–13]. There were 167 patients originally randomized into the PTAE arm; however, 40 patients did not receive PTAE (23.9%) [8, 18]. Seven out of 14 (50.0%) rebleeds and 4 out of 5 (80.0%) deaths were from this subgroup [8, 18]. The lack of radiological resources was the most common cause (34.3%) [A1] followed by the inability to catheterize the targeted vessel (28.6%, Fig. 2). Because of this subgroup’s potential impact on the intention-to-treat analysis as suggested by Loffroy et al. [14], only patients from per-protocol-analyses of these two studies were considered in the following meta-analysis.

**Table 1:** Baseline characteristics and outcomes of the included studies. *ITT* intention-to-treat, *EO* endoscopy only group, *PPA* per-protocol-analysis, *PTAE* prophylactic embolization group, *RC* retrospective cohort study, *RCT* randomized-controlled trial, *NS* not specified

Study	Country	Design	Sample size	Follow-up period	Forrest class	PTAE indication
Lau 2019 [8]	China	RCT	EO, 123 PTAE, 96 (PPA), 118 (ITT)	30 days	Ia, 38 (17.4%) Ib, 82 (37.4%) IIa, 99 (45.2%)	Spurting hemorrhage during endoscopy; ulcers ≥ 20 mm; hemoglobin < 9 g/dL on admission; systolic pressure < 90 mmHg and pulse > 110 beats/min
Laursen 2014	Denmark	RCT	EO, 56 PTAE, 31 (PPA), 49 (ITT)	30 days	Ia, 11 (10%) Ib, 34 (32%) IIa, 53 (50%) IIb, 7 (7%)	Forrest class I-IIb
Mille 2015 [13]	Germany	RC	EO, 47 PTAE, 55	30 days	Ia, 19 (16%) Ib, 39 (33%) IIa, 14 (12%) IIb, 10 (9%) IIc, 8 (7%) III, 27 (23%)	Ulcer located in the posterior duodenal bulb; Forrest I-IIc with a Rockall Score ≥ 6; active bleeding or ulcer ≥ 1 cm on endoscopy; systolic pressure < 100 mm Hg, age > 80 years, ≥ 2 anticoagulants, ≥ 2 comorbidities
Kamiski 2017	Latvia	RC	EO, 50 PTAE, 25	NS	Ia, 16 (21.3%) Ib, 11 (14.7%) IIa, 33 (44.0%) IIb, 15 (20.0%)	Forrest I-IIb and Rockall Score ≥5
Kamiski 2019	Latvia	RC	EO, 341 PTAE, 58	NS	Ia, 46 (11.5%) Ib, 61 (15.3%) IIa, 104 (26.1%) IIb, 188 (47.1%)	Forrest I-IIb and Rockall Score ≥5
Total			EO, 617 PTAE, 265

### Rebleeding

**Fig. 3:**
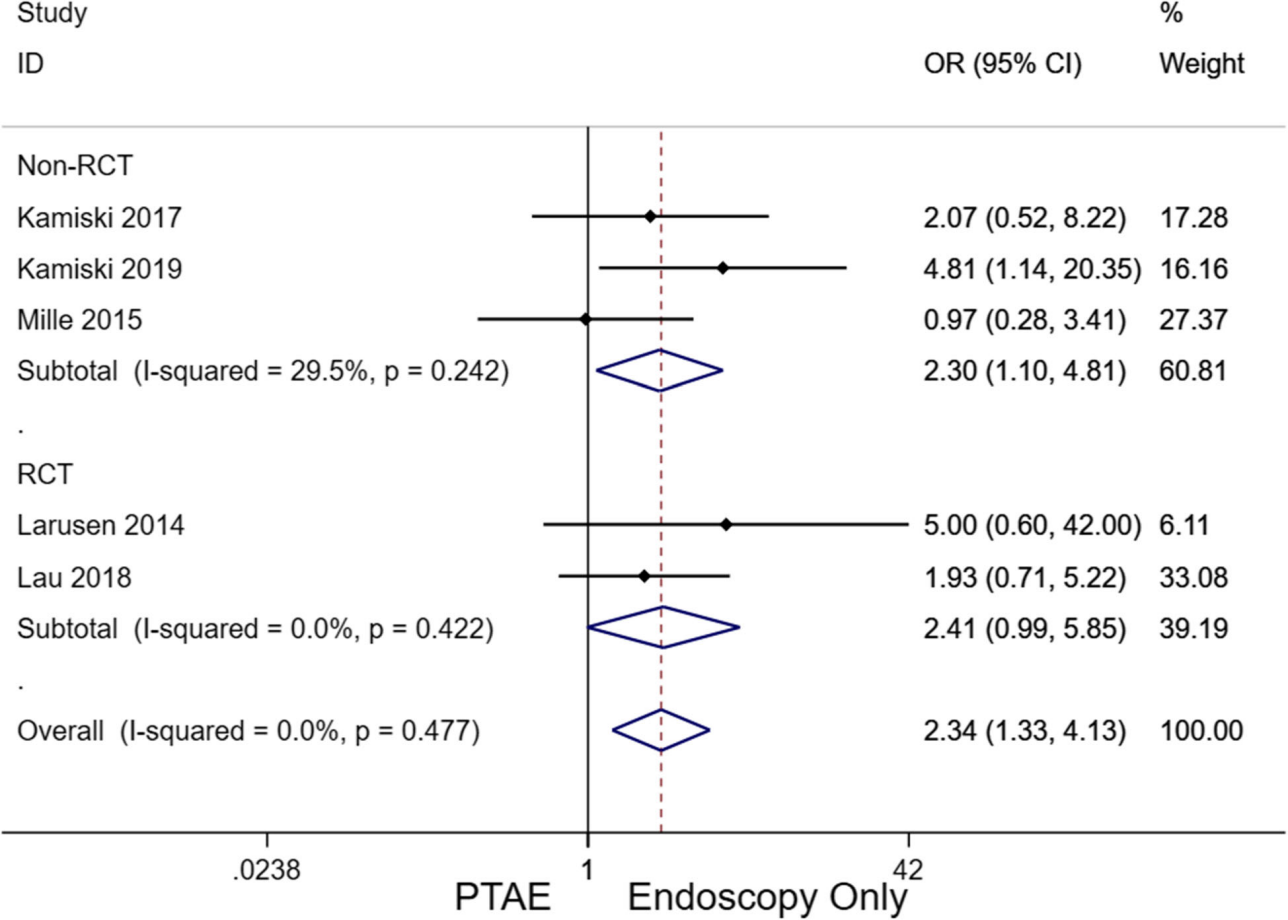
Forest plot for OR comparing rebleeding between PTAE and EO groups (overall, 2.34 [95% CI, 1.33–4.13], *p* = 0.003). A fixed-effect model was implemented (I^2^ = 0.0%). CI, confidence interval; EO, endoscopy only; OR, odds ratio; PTAE, prophylactic transcatheter embolization

All five studies reported the risk of rebleeding as follows: 18/265 (6.8%) patients in the PTAE group and 88/617 (14.3%) patients in the EO group (Supplement Table 1). The odds ratio (OR) of rebleeding was significantly higher in patients without additional PTAE, 2.34 [95% CI, 1.33–4.13], *p* = 0.003 (Fig. 3). Subgroup analysis according to study type was consistent with the overall findings, 2.304 [95% CI, 1.10–4.81] in retrospective cohort studies (*p* = 0.026) and 2.41 [95% CI. 0.99–5.85] (*p* = 0.053) in RCT. The borderline lack of significance among RCTs was largely due to the lack of statistical power.

### Need for surgery

**Fig. 4:**
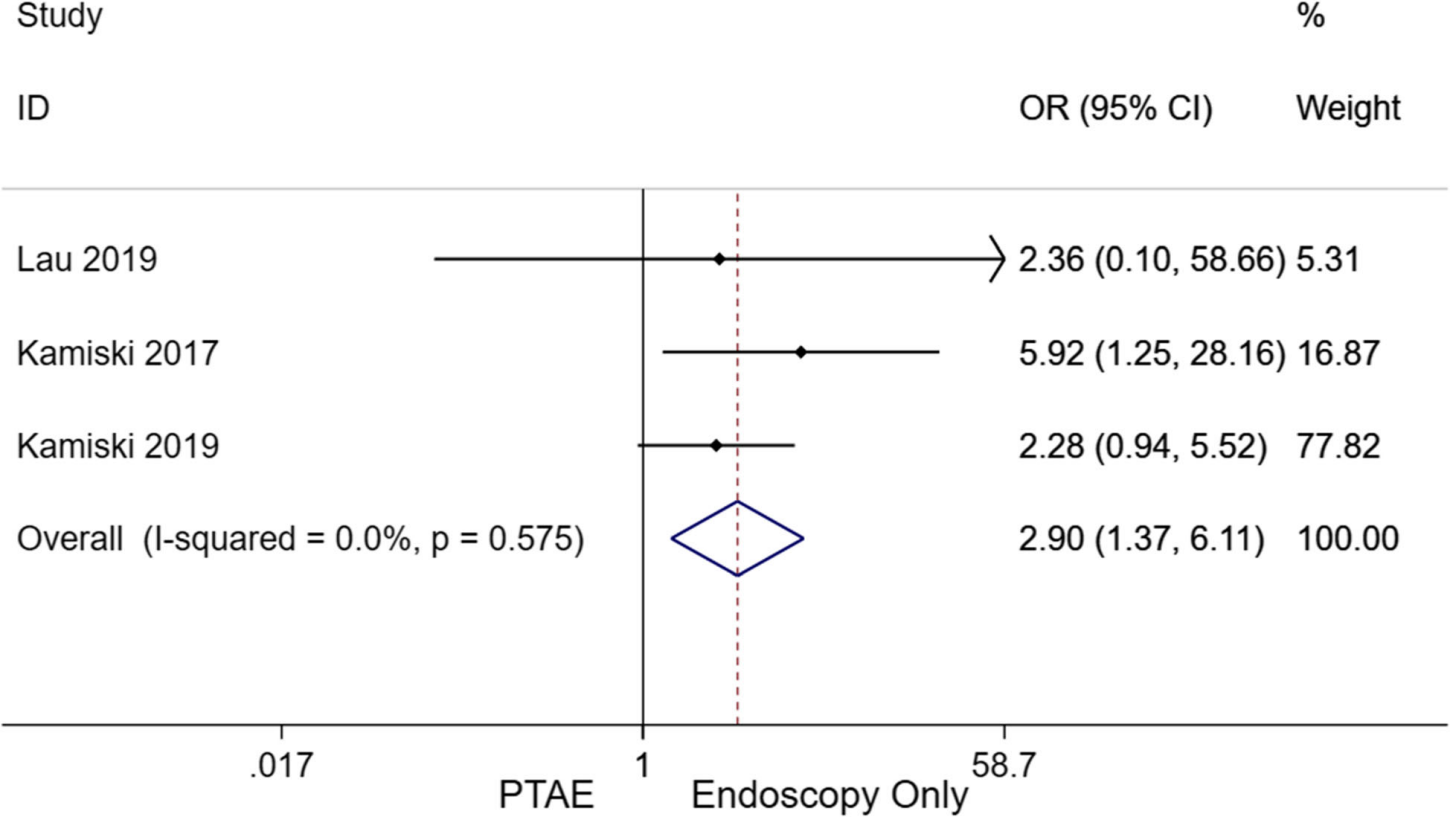
Forest plot for OR comparing the need of surgery between PTAE and EO groups (OR, 2.898; 95% CI [1.374–6.112], *p* = 0.005). A fixed-effect model was implemented I^2^ = 0.0%. CI, confidence interval; EO, endoscopy only; OR, odds ratio; PTAE, prophylactic transcatheter embolization

In total, 89 patients from the EO group eventually required surgical intervention to control rebleeding (14.4%) compared to eight patients from the PTAE group (3.0%, Supplement Table 2). Overall, EO had a significantly higher need for surgery than PTAE (Fig. 4; OR, 2.898; 95% CI [1.374–6.112], *p* = 0.005). Three of the five studies reported no need for surgical intervention among PTAE patients, including both RCTs and 1 RC (Supplement Table 2).

### Mortality

**Fig. 5:**
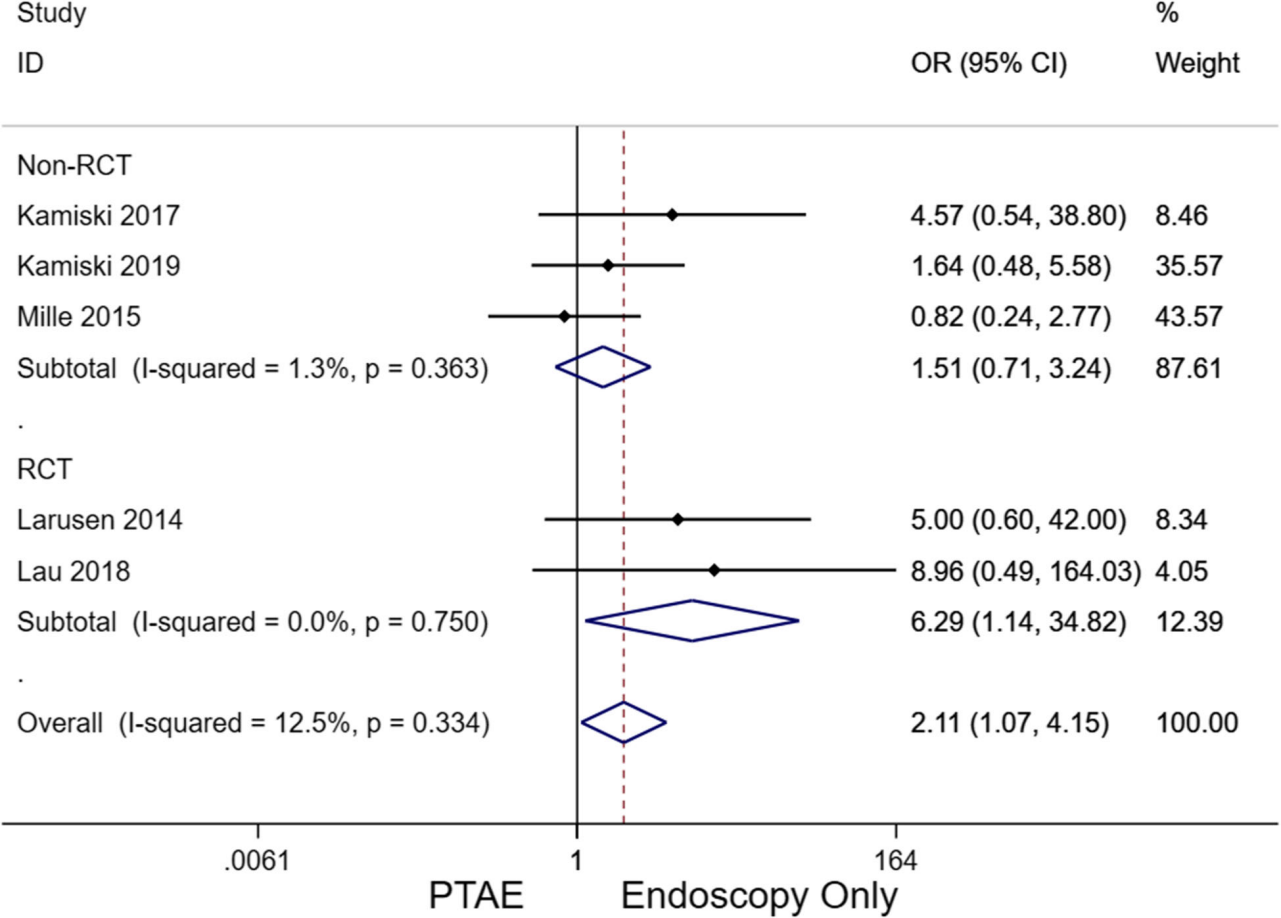
Forest plot for OR comparing mortality between PTAE and EO groups (overall, 2.11; 95% CI [1.07–4.15], *p* = 0.032). A fixed-effect model was implemented (I^2^ = 12.5%). CI, confidence interval; EO, endoscopy only; OR, odds ratio; PTAE, prophylactic transcatheter embolization

Among five studies, 12/265 (4.5%) and 54/617 (8.8%) patients from the PTAE and EO groups, respectively, died during follow-up assessments (Supplement Table 3, Fig. 5). Overall, EO had a significantly higher mortality rate than PTAE (OR, 2.11; 95% CI [1.07–4.15], *p* = 0.032), which also held true for the RCT subgroup (OR, 6.294; 95% CI [1.14–34.82], *p* = 0.035).

### Complication rate

According to the Quality Improvement Guidelines for percutaneous transcatheter embolization per the Society of Interventional Radiology [19], nine patients encountered minor complications that did not require further intervention (3.4%), eight of which were asymptomatic (Table 2). Only two major complications occurred: 1 pancreatitis and 1 hepatic failure (0.75%). All complications were due to coil migration.

**Table 2:** Complications from prophylactic transcatheter embolization

Study	Embolization-related complication
Lau 2019 [8]	None
Laursen 2014	1 coil migration, asymptomatic
Mille 2015	8 minor complications; 2 major complications.
Kamiski 2017	None
Kamiski 2019	None
Total	Minor, 9/265 (3.4%) Major, 2/265 (0.75%)

### Publication bias and sensitivity analysis

**Fig. 6:**
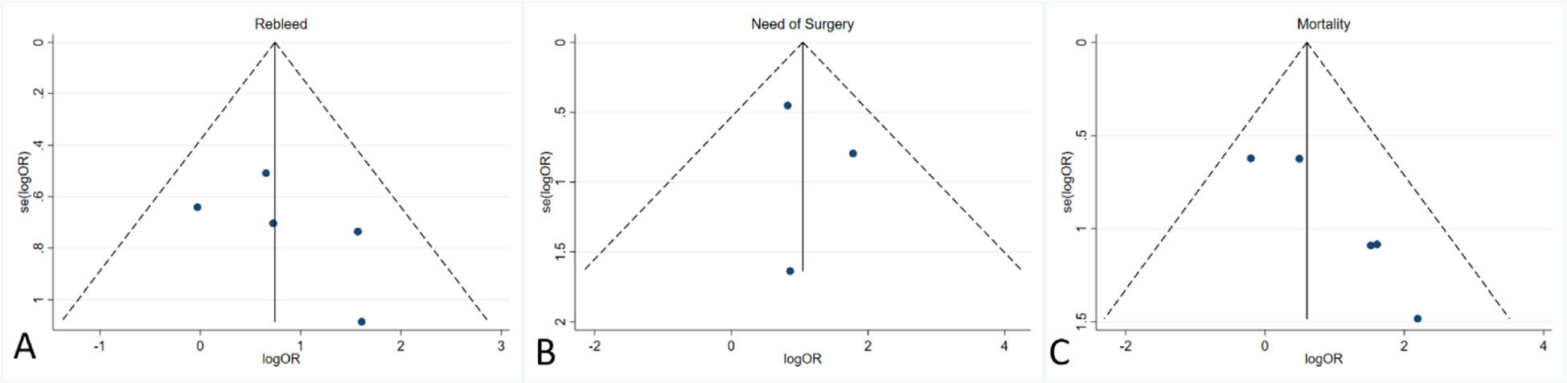
Funnel plots were implemented to evaluate publication bias. **A** Odds ratio (OR) of rebleeding after prophylactic transcatheter embolization (PTAE) and endoscopy only (EO). **B** OR of surgical requirement after PTAE and EO. **C** OR of mortality after PTAE and EO

Funnel plots (Fig. 6) and Egger’s tests were implemented to evaluate publication bias. In terms of rebleeding rates and need for surgery, no publication bias was appreciated according to funnel plot (Fig. 6) and Egger’s test (*p* = 0.336 and 0.739). In terms of mortality rates, the funnel plot suggested possible asymmetry (Fig. 6); Egger’s test was significant for publication bias (*p* = 0.028). The results of the metaninf analysis indicated that the elimination of studies might affect the final results (Supplement Fig. 1.1, 1.2; Supplement Table 4.1, 4.2).

### Subgroup analysis of clinically relevant variables

Subgroup analysis was performed based on the follow-up duration, hemoglobin level at presentation, and non-aspirin NSAID use (Table 3). PTAE has significantly lower rates of rebleeding and mortality in patient cohorts with greater than 20% NSAID [A3] and hemoglobin levels greater than 8.0g/dL. At 30-day follow-up, neither mortality nor rebleeding rate was statistically significant in terms of PTAE’s efficacy over conservative management after endoscopy.

**Table 3:** Subgroup analysis of the efficacy of prophylactic transcatheter embolization in nonvariceal upper gastrointestinal bleeding; *OR* odds ratio

Clinical variable	Study number	Rebleed		Mortality	
		OR (95% CI)	*P* value	OR (95% CI)	*P* value
Hemoglobin (mean or median)					
< 8.0	3^c,d,e^	1.495 (0.694–3.219)	0.305	1.458 (0.545–3.897)	0.452
≥ 8.0	2^a,b^	3.642 (1.491–8.895)	0.005	8.77 (3.344–22.997)	< 0.001
Percentage of NSAID use (excluding aspirin)					
≥ 20%	2^c,d^	2.83 (0.91–8.87)	0.074	8.0 (1.753–36.512)	0.007
< 20%	2^a,b^	1.49 (0.69–3.22)	0.305	1.458 (0.545–3.897)	0.452
Follow-up					
30-day	3^a,b,c^	1.816 (0.896–3.683)	0.098	2.209 (0.94–5.192)	0.069
NS	2^d,e^	3.394 (1.268–9.082)	0.015	8.981 (3.05–26.439)	< 0.001

^a^Mille 2015

^b^Lau 2018 [8]

^c^Laursen 2014

^d^Kamiski 2017

^e^Kamiski 2019

## Discussion

Transcatheter arterial embolization (TAE) is widely acknowledged for its effectiveness in treating brisk arterial bleeding [20]. It has largely replaced surgery as a first-line therapy for UGIB refractory to endoscopic treatment due to its high efficacy and favorable safety profile [21]. However, a subgroup of ulcers remains at high risk for future rebleeding despite achieving hemostasis via endoscopy. These high-risk lesions are typically large in size (i.e., greater than 10mm), belong to Forrest class I-IIb, and/or were treated in hemodynamically unstable patients [8, 13, 22]. While residual blood flow underneath a treated lesion may serve as a source of rebleeding, the intermittent nature of UGIB is dependent on a patient’s hemodynamic status. For example, a hypotensive patient may present with a lesion that appeared hemostatic at endoscopy; however following blood transfusion, the normalized blood pressure could initiate rebleeding [23–25]. In 2012, based on the available data at that time, the American College of Gastroenterology Guidelines only recommended medical treatment of the underlying etiology such as withholding aspirin, treating *H. pylori*, etc. Studies have been published in the last 5 years showing the use of PTAE to decrease this risk and mortality in selected patients [8, 26]. As such, most authors considered PTAE as an option if the ulcer was deemed high risk based on endoscopic findings (i.e., large ulcers, Forrest class I-IIb) and/or clinical presentation (i.e., hypotension, decreased hemoglobin during endoscopy) [8, 11–13, 18]. Mille et al. considered PTAE in patients with Forrest class I-IIc ulcers based on the presence of clinical and individual risk factors [13]. Twenty-seven patients from the Mille et al. study were evaluated to be Forrest class III; however, none received PTAE and were instead treated with proton pump inhibitors and/or EO [13].

According to the present meta-analysis, PTAE was highly effective in reducing the risk of rebleeding compared to EO (2.34 [95% CI, 1.33–4.13], *p* = 0.003). Compared to patients who did not receive additional embolization after endoscopy, the PTAE group had a statistically significantly lower rebleeding rate of 6.8%. This value was also lower than the reported rates of emergent embolization for nonvariceal UGIB, which ranged from 9 to 47% [27–31]. For nonvariceal UGIB recalcitrant to TAE, surgery is considered to be the final definitive treatment option. However, surgery confers a mortality risk as high as 43% [32, 33]. Three out of the five included study cohorts required surgical intervention, with a higher cumulative rate in patients that did not receive PTAE after endoscopy (14.4% vs 3.0%; OR, 2.898; 95% CI [1.374–6.112], *p* = 0.005). Further, the PTAE group also had a statistically significantly lower mortality rate than its EO counterpart (4.5% vs 8.8%, OR, 2.11; 95% CI [1.07–4.15], *p* = 0.032), though publication bias exists (Fig. 6C). A total of twelve PTAE patients from four studies died. According to Laursen et al., the majority of deaths were non-UGIB related (5/7), such as sepsis and malignancy [18]. In one case, an endovascular coil migrated to the hepatic artery, leading to acute hepatic failure and death. Of note, this patient had poor baseline liver function at admission due to advanced cirrhosis. Isolated coil migration to the hepatic artery is not expected to routinely cause fulminant hepatic failure due to the dual blood supply of the liver. Coil migration also occurred in nine other patients, which were all asymptomatic without elevation of liver enzymes. Eight of these nine patients were from the same study, so this type of complication may have been operator dependent. Nevertheless, only two studies observed complications from PTAE with a major and minor complication rate of 0.75% and 3.4%, respectively [13, 18]. In comparison to the differences of mortality and rebleed rates between PTAE and EO groups, the risk of having a major PTAE-related complication was considered rather minimal. Based on subgroup analysis (Table 3), patients using NSAIDs are more likely to benefit from PTAE because of the higher rebleeding rates in this population [34]. In terms of baseline hemoglobin level, it was interesting to see that patients with a lower hemoglobin level are less likely to benefit from additional PTAE (Table 3). The absolute rebleeding and mortality rates between lower and higher hemoglobin subgroups were comparable in the PTAE arm (4.6% [7/151] vs 4.4%[5/114]) and mortality (7.9%[12/151] vs 5.3%[6/114]); by contrast, the rebleeding and mortality rates were contra-intuitively higher among patients with higher hemoglobin levels in the EO arm (rebleed, 15.4% vs [69/447] vs 11.2% [19/170]; mortality, 9.8% [44/447] vs 5.9% [10/17]), contributing to the observed more prominent effect of PTAE in patients with higher hemoglobin. Of note, these subgroup analysis results were based on few study-level evidence, which subjects to confounding factors and should be interpreted with caution. Stratified analysis using patient-level data are warranted to investigate the relationship between hemoglobin level and the effect of PTAE in future studies.

There are several limitations to this study. Firstly, only five studies were included, three of which were retrospective cohorts, which are inferior to RCT and prospective studies. Due to limited evidence available, study removal during sensitivity analysis could render the overall results less statistically significant (Supplement Fig. 1.1, 1.2). Secondly, the length of follow-up time varied among studies. Subgroup analysis on 30-day follow-up did not suggest a statistically significant advantage of PTAE in terms of rebleeding and mortality reduction, despite the cumulative overall results suggesting its efficacy (Table 3). The rebleeding and mortality rates are expected to increase cumulatively over time. Only Lau et al. implemented a Kaplan-Meier curve to characterize these evolutions [8]. By contrast, the two studies that did not specify follow-up outcomes demonstrated a combined result favoring PTAE (Table 3). Whether the follow-up period is shorter or longer than 30 days affects our interpretation of PTAE’s advantage in short versus long-term follow. Finally, limited evidence was available regarding the risk factors of rebleeding and deaths after PTAE. Subgroup analysis of additional factors such as Forrest Classification, Rockall score, ASA Physical Classification, and anticoagulation use (warfarin, heparin) were not possible, as original studies did not compare clinical outcomes between groups with and without PTAE stratified based on these variables.

Given these limitations, several important aspects should be addressed during future study design. In addition to increasing sample size, future RCTs with intention-to-treat protocols should aim to reduce the number of patients allocated to PTAE that fail to receive the procedure. For example, increased access to centers with expert resources in interventional radiology or modification of protocols to conduct angiography immediately following endoscopy should be considered. Further, standardization of technique is also important with respect to embolic(s) used and method of embolization. As suggested by Loffroy et al., variations may contribute to rebleeding risk, and few authors from prior literature have specified procedure details [14, 27]. Finally, stratification based on lesion type and size should also be performed. The post hoc analysis by Lau et al. suggested that PTAE reduced rebleeding risk in ulcers greater than 15mm [8]. Additional clinical trials with long-term clinical outcomes are needed to confirm these observations.

## Conclusion

PTAE compared to observation alone appears to safely reduce the risk of rebleeding, need for surgical intervention, and morality in high-risk peptic ulcers after successful endoscopic hemostasis. Future studies should aim to increase the sample size and resources for performing endovascular interventions.

## Supplementary Information

**Additional file 1: S. Table 1.** Rebleeding rate between EO (14.3%) and PTAE (6.8%) groups. EO: endoscopy only. PTAE: prophylactic transcatheter embolization. **S. Table 2.** Pooled rate of surgical intervention after EO (86/617, 14.4%) or PTAE (8/265, 3%). EO: endoscopy only. PTAE: prophylactic transcatheter embolization. **S. Table 3.** Mortality rate between EO (8.8%) and PTAE (4.5%) groups. EO: endoscopy only. PTAE: prophylactic transcatheter embolization. **S. Table 4.1.** Sensitivity Analysis of the odds ratio of rebleeding risk after PTAE versus conservative management. **S. Figure 1.1.** Sensitivity Analysis of the odds ratio of rebleeding risk after PTAE versus conservative management. **S. Table 4.2.** Sensitivity Analysis of the odds ratio of mortality after PTAE versus conservative management. **S. Figure 1.2.** Sensitivity Analysis of the odds ratio of rebleeding risk after PTAE versus conservative management. **Supplement Table 5.1.** Newcastle Ottawa Scale (NOS) of cohort studies was used to evaluate the quality for each eligible study. **Supplement Table 5.2.** The Cochrane Collaboration’s tool for assessing risk of bias in randomized trials.

## Data Availability

The datasets during and/or analyzed during the current study are available from the corresponding author on reasonable request.

## Abbreviations

EO: Endoscopy only
PTAE: Prophylactic transarterial embolization
RC: Retrospective cohort
RCT: Randomized-controlled trial
TAE: Transcatheter arterial embolization
UGIB: Upper gastrointestinal bleed
