# Supplementary material for: Prophylactic transcatheter arterial embolization for high-risk ulcers following endoscopic hemostasis: a meta-analysis
Source: World J Emerg Surg. 2021 Jun 10;16:29. doi: 10.1186/s13017-021-00371-2 (PMC8194167; doi:10.1186/s13017-021-00371-2)
Supplement: Supplementary file 1 — Additional file 1: S. Table 1. Rebleeding rate between EO (14.3%) and PTAE (6.8%) groups. EO: endoscopy only. PTAE: prophylactic transcatheter embolization. S. Table 2. Pooled rate of surgical intervention after EO (86/617, 14.4%) or PTAE (8/265, 3%). EO: endoscopy only. PTAE: prophylactic transcatheter embolization. S. Table 3. Mortality rate between EO (8.8%) and PTAE (4.5%) groups. EO: endoscopy only. PTAE: prophylactic transcatheter embolization. S. Table 4.1. Sensitivity Analysis of the odds ratio of rebleeding risk after PTAE versus conservative management. S. Figure 1.1. Sensitivity Analysis of the odds ratio of rebleeding risk after PTAE versus conservative management. S. Table 4.2. Sensitivity Analysis of the odds ratio of mortality after PTAE versus conservative management. S. Figure 1.2. Sensitivity Analysis of the odds ratio of rebleeding risk after PTAE versus conservative management. Supplement Table 5.1. Newcastle Ottawa Scale (NOS) of cohort studies was used to evaluate the quality for each eligible study. Supplement Table 5.2. The Cochrane Collaboration’s tool for assessing risk of bias in randomized trials. [file 13017_2021_371_MOESM1_ESM.docx]

**SUPPLEMENT**

| Study | Rebleeding Rate in EO Group | Rebleeding Rate in PTAE Group |
| --- | --- | --- |
| Lau 2019 | 14/123 | 6/96 |
| Laursen 2014 | 8/56 | 1/31 |
| Mille 2015 | 5/47 | 6/55 |
| Kamiski 2017 | 11/50 | 3/25 |
| Kamiski 2019 | 50/341 | 2/58 |
| Total | 88/617 (14.3%) | 18/265 (6.8%) |

**S. Table 1**: Rebleeding rate between EO (14.3%) and PTAE (6.8%) groups. EO: endoscopy only. PTAE: prophylactic transcatheter embolization

| Study | Need of Surgical Intervention in EO Group | Need of Surgical Intervention in PTAE Group |
| --- | --- | --- |
| Lau 2019 | 1/123 | 0/96 |
| Laursen 2014 | 0/56 | 0/31 |
| Mille 2015 | 0/47 | 0/55 |
| Kamiski 2017 | 17/50 | 2/25 |
| Kamiski 2019 | 71/341 | 6/58 |
| Total | 86/617 (14.4%) | 8/265 (3.0%) |

**S. Table 2**: Pooled rate of surgical intervention after EO (86/617, 14.4%) or PTAE (8/265, 3%). EO: endoscopy only. PTAE: prophylactic transcatheter embolization.

| Study | Mortality in EO Group | Mortality in PTAE Group |
| --- | --- | --- |
| Lau 2019 | 5/123 | 0/96 |
| Laursen 2014 | 8/56 | 1/31 |
| Mille 2015 | 5/47 | 7/55 |
| Kamiski 2017 | 8/50 | 1/25 |
| Kamiski 2019 | 28/341 | 3/58 |
| Total | 54/617 (8.8%) | 12/265 (4.5%) |

**S. Table 3**: Mortality rate between EO (8.8%) and PTAE (4.5%) groups. EO: endoscopy only. PTAE: prophylactic transcatheter embolization.

| Study Omitted | Estimate | 95% Confidence Interval |
| --- | --- | --- |
| Mille 2015 | 2.8606365 | 1.4813247 - 5.5242724 |
| Lau 2018 | 2.5500574 | 1.2757051 - 5.0974102 |
| Laursen 2014 | 2.1709063 | 1.2014346 - 3.9226725 |
| Kamiski 2017 | 2.401356 | 1.2882692 - 4.4761691 |
| Kamiski 2019 | 1.8683306 | .99629974 - 3.5036235 |
| Combined | 2.3438137 | 1.328911 - 4.133808 |

**S. Table 4.1**: Sensitivity Analysis of the odds ratio of rebleeding risk after PTAE versus conservative management.


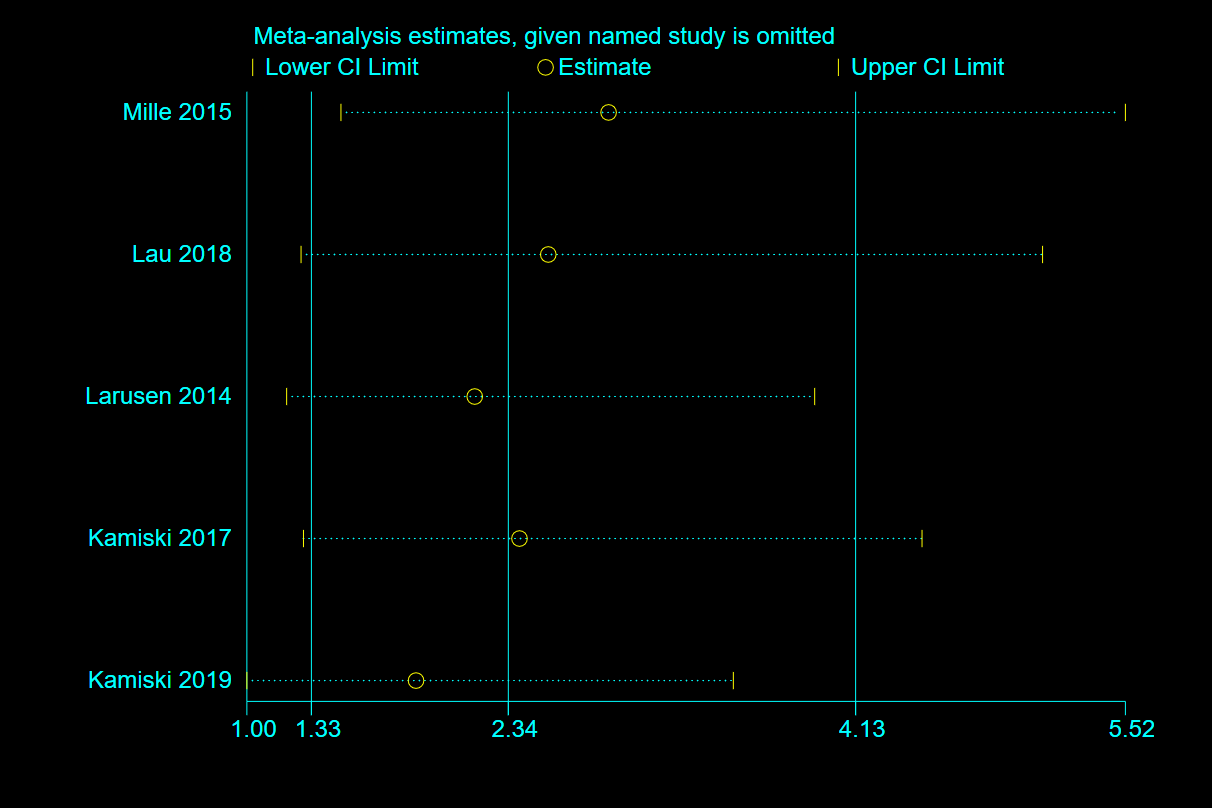


**S. Figure 1.1**: Sensitivity Analysis of the odds ratio of rebleeding risk after PTAE versus conservative management.

| Study Omitted | Estimate | 95% Confidence Interval |
| --- | --- | --- |
| Mille 2015 | 3.1018512 | 1.2680222 - 7.5877862 |
| Lau 2018 | 1.8166653 | .89489925 - 3.6878705 |
| Laursen 2014 | 1.8427566 | .89437312 - 3.7967956 |
| Kamiski 2017 | 1.8780748 | .91301966 - 3.8631861 |
| Kamiski 2019 | 2.3633807 | 1.0529445 - 5.3047128 |
| Combined | 2.1060643 | 1.0676613 - 4.1544137 |

**S. Table 4.2**: Sensitivity Analysis of the odds ratio of mortality after PTAE versus conservative management.


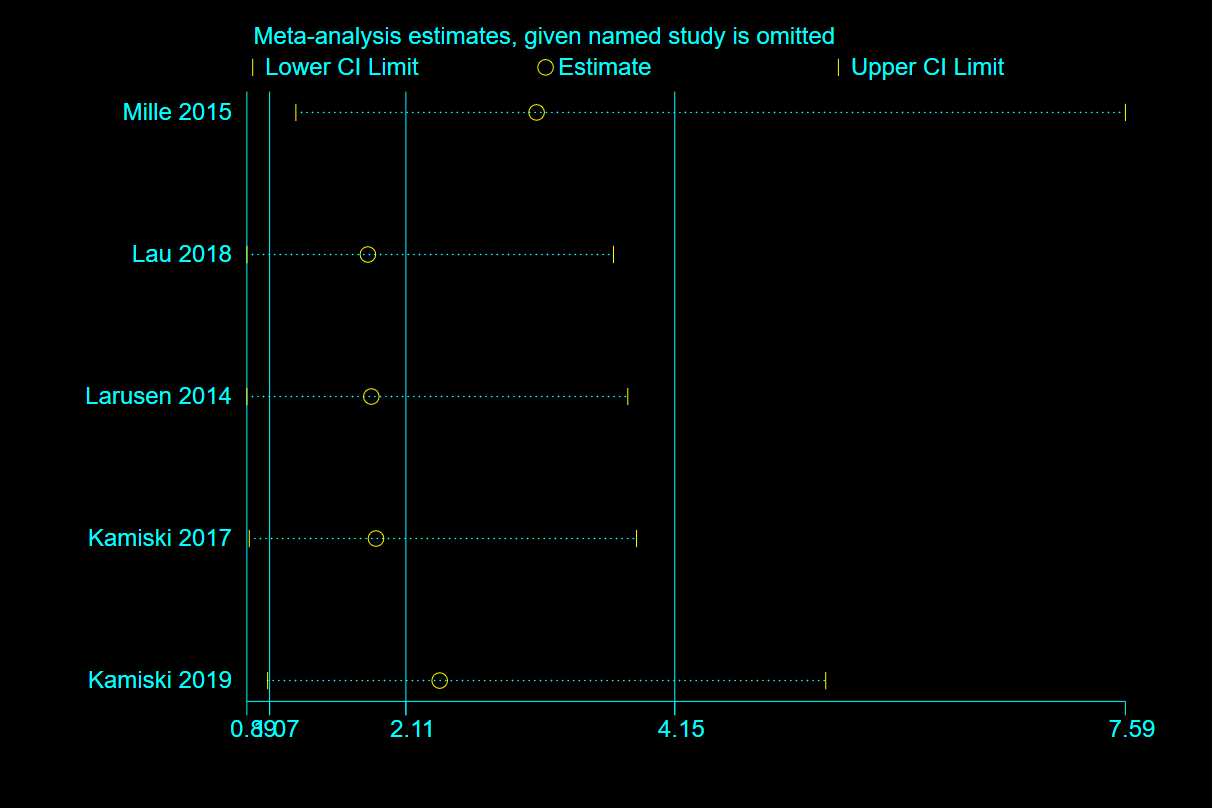


**S. Figure 1.2**: Sensitivity Analysis of the odds ratio of rebleeding risk after PTAE versus conservative management.

| **Study** | **Selection** | | | | **Comparability** | **Outcome** | | | **Overall** |
| --- | --- | --- | --- | --- | --- | --- | --- | --- | --- |
|  | **Representativeness of the exposed cohort** | **Selection**  **of the non-exposed cohort** | **Ascertainment**  **Of**  **Exposure** | **Outcome of**  **Interest was**  **not present at start of study** | **Comparability of Cohorts on the Basis of the Design or Analysis** | **Assessment of**  **Outcome** | **Follow-Up Long Enough for Outcomes to Occur** | **Adequacy of Follow Up of Cohorts** |  |
| Mille 2005 | ★ | ★ | ★ | ★ | ★☆ | ★ | ★ | ★ | **8** |
| Kamiski 2017 | ★ | ★ | ★ | ★ | ★☆ | ★ | ★ | ☆ | **7** |
| Kamiski 2019 | ★ | ★ | ★ | ★ | ★☆ | ★ | ★ | ☆ | **7** |

**Supplement Table 5.1: Newcastle Ottawa Scale (NOS) of cohort studies was used to evaluate the quality for each eligible study.**

| Study | **Selection Bias** | | **Performance Bias** | **Detection Bias** | **Attrition Bias** | **Reporting Bias** | **Other Bias** | **Total** |
| --- | --- | --- | --- | --- | --- | --- | --- | --- |
|  | Random Sequence Generation | Allocation Concealment | Blinding of Participants and Personnel | Blinding of Outcome Assessment | Incomplete Outcome Data | Selective Reporting | Many patients allocated to the treatment group did not end up receiving the treatment | Low on Risk of Bias |
| Lau | Low | High | High | High | Low | Low | High | 3/7 |
| Laursen | Low | High | High | High | Low | Low | High | 3/7 |

**Supplement Table 5.2: The Cochrane Collaboration’s tool for assessing risk of bias in randomized trials.**
